# No age thresholds in the emergency department: A retrospective cohort study on age differences

**DOI:** 10.1371/journal.pone.0210743

**Published:** 2019-01-30

**Authors:** Caro Fuchs, Bilge Çelik, Steffie H. A. Brouns, Uzay Kaymak, Harm R. Haak

**Affiliations:** 1 Eindhoven University of Technology, School of Industrial Engineering, Eindhoven, The Netherlands; 2 Department of Internal Medicine, Máxima Medical Center, Eindhoven, the Netherlands; 3 Department of Internal Medicine, Division of General Internal Medicine, Maastricht University Medical Centre+, Maastricht, the Netherlands; 4 Ageing and Long-Term Care, CAPHRI School for Public Health and Primary Care, Maastricht University, Maastricht, the Netherlands; Medical University Graz, AUSTRIA

## Abstract

Emergency care in elderly patients has gained attention by researchers due to high utilization rate and the importance of emergency services in elderly care. We examine if there is a clear age threshold between young and old patients at which there is a need for extra care and facilities in the emergency department. This retrospective cohort study uses emergency department (ED) data collected over the course of a year, containing information about 31,491 patient visits. The measured variables are treatment time, waiting time, number of tests, number of medical procedures, number of specialties involved and the patient’s length of stay on the ED. To examine the multivariate differences between different patient groups, the data set is split into eighteen age groups and a MANOVA analysis is conducted to compare group means. The results show that older patients tend to have a longer stay on the ED. They also require more medical tests, have higher resource utilization and admission rates to the hospital. When the patients are grouped according to life stages (<18, 18-39, 40-64 and ≥65), each life stage shows significantly different characteristics across all variables. To understand where these differences start, age bins of five years are analyzed and almost none of the consecutive groups are significantly different in any variable. A significant difference between all groups is observed when age interval of the bins is increased to 10 years. This indicates that although age has an effect on the patient’s treatment, a clear age threshold that identifies the group of elderly patients is not observable from emergency room variables. The results of this study show no clear age boundary between young and old patients. In other words, we could not find support for favoring the often-used age boundary of 65 over other boundaries (e.g. 60 or 70) to distinguish the group of elderly patients on the ED.

## Introduction

In 2017, there are an estimated 962 million people aged 60 or over in the world, comprising 13 per cent of the global population, and the population aged 60 or over keeps growing at a rate of about 3 per cent per year [1]. The treatment in the emergency department has a critical role in the elderly care, and successful treatment of acute failures is enabled by identifying the requirements of elderly patients accurately [2].

Previous studies suggest that patients older than 75 years use the hospital’s emergency department (ED) twice as much as 65-75 years old [3]. Patients older than 65 years use the ED twice as often as patients younger than 65 [4] years do. Compared to younger patients, older patients tend to develop a more severe disease state and have co-morbidities [5] leading to a longer stay on the ED, [3, 5–12] have more tests and resource utilization [2, 5, 6, 9, 11, 13, 14] and higher admission rates to the hospital [2, 5, 6, 8, 12, 14–17].

Elderly have different needs compared to other patients. Understanding these differences is crucial to delivering the optimal care to these patients. In the management of acute health care, age thresholds are used or discussed in respect of risk for adverse events and need for extra care (for example in [18]). Identifying age threshold at which there is such a need for extra care and facilities would be useful for planning and management of the acute care. In this analysis, we sought to determine if there is a clear age threshold between young and old patients at which there is a need for extra care and facilities in the emergency department.

## Methods

### Study design and setting and selection of participants

This retrospective cohort study is based on data obtained from the Máxima Medical Centre’s Emergency Department. Máxima Medical Centre is a teaching hospital with urban and regional function in the south of the Netherlands. The majority of ED patients was referred by a general practitioner (GP) or came by self-referral. Other modes of presentation were by the referral of a medical specialist and ambulance arrival in high emergency patients.

Triage at presentation was performed using the Manchester Triage System (MTS) [19], a five-level system (red, orange, yellow, green, and blue). Patients presenting to the ED were assessed by a medical student in the last year of medical education, a resident or an emergency physician, supervised by a medical specialist [20].

The Ethical Review Board of the Máxima Medical Centre decided that the study did not fall under the scope of the Medical Research Involving Human Subjects Act (WMO) because of its retrospective nature. All data were anonymous to the researchers.

### Data collection and processing

Data on all ED visits were retrospectively collected over the course of a year, from the 1st of September 2010 until the 31st of August 2011. Data were extracted according to a fixed data collection form by one abstractor with a medical background, who was blinded to the study hypothesis. ED visits were selected for inclusion in this study if the patient underwent diagnostic tests or treatment on the ED. Therefore, data about patients who only used the ED as an entrance to the hospital and were directly transferred to other departments (e.g. the cardiac care unit) were excluded (N = 3696). To identify these patients, the variable ‘length of stay’ (LOS) was used (cases with LOS = 0.00 were excluded). Another exclusion criterion was patients presenting with bite and needle stick injuries (N = 114) since these patients are hospital personnel, who are required to report for examination after such injuries.

Baseline and medical data were retrospectively gathered from electronic patient records. Data included age, gender, type and number of diagnostic tests (laboratory test, glucose measurement, arterial blood gas, urine analysis, blood culture, sputum culture, X-ray, ultrasonography, computed tomography scan and electrocardiography), and medical procedures (intubation, placement of urinary catheter or gastric tube, cardiac rhythm monitoring, administration of oxygen, intravascular cannula, vital functions monitoring, wound dressing, pressure bandage, plaster cast, sling, administration of anti-tetanus immunoglobulin, tetanus toxoid vaccination, intubation, gavage and eye bandage) performed on the ED. Other measured variables were triage level, the treatment time, waiting time, length of stay, medication administered on the ED, number of specialties involved with treatment and the patient’s length of stay on the ED.

Triage at presentation was performed using the MTS. Urgency levels were categorised as 1-4 (respectively red, orange, yellow and green). Category blue is not used on the ED. Type of referral was divided into referral by a GP, ambulance, medical specialist or self-referral. Waiting times were defined as the time from ED arrival to ED bed placement. Treatment times were the time from ED bed placement to final disposition. The length of stay was defined as the time between ED arrival and ED discharge or hospital admission. ED visits were divided into medical (internal medicine, pulmonology, cardiology, neurology, psychiatry, rheumatology, gastroenterology, and paediatrics), or trauma (general surgery, plastic surgery, orthopaedics, gynaecology, ENT, ophthalmology, dermatology) visits. The destination was discharge home with or without follow-up, admission to the acute admission unit (AAU), admission to another ward, and mortuary.

### Outcome measurements

The primary endpoint of interest was the identification of an age threshold between young and old patients at which there is a need for extra care and facilities in the emergency department. Patients were categorized according to life stages (<18, 18-39, 40-64 and ≥65) and as elderly or non-elderly with various cut off points to see differences between predefined age categories. Then, age was divided in eighteen groups: 0-5, 6-10, 11-15, 16-20, 21-25, 26-30, 31-35, 36-40, 41-45, 46-50, 51-55, 56-60, 61-65, 66-70, 71-75, 76-80, 81- 85, ≥86 year. The five year age intervals were chosen as the minimum age groups that gives balanced group sizes as an initial step to identify when the significant differences occur between age groups. Evaluation of differences in emergency care was based on the triage level, number of diagnostic tests, number of medical procedures performed on the ED, number of specialties involved, treatment time, waiting time and length of stay.

### Statistical analysis

Statistical analysis was performed using IBM SPSS for Mac, version 22 (IBM Corp. Armonk, NY). Some of the variables in the data set were not normally distributed, so Tukey’s outlier labeling rule was used for detecting univariate outliers [19, 21, 22]. For detecting multivariate outliers, the Mahalanobis distance was used. Outliers were recorded as missing values since the values showed hints of recording errors with unreasonable values. To analyze the underlying structure of missingness, Little’s MCAR test [23] and independent t-tests are used to compare missing and complete instances.

Age was evaluated as a categorical variable in all analyses to allow detection of nonlinear or threshold effects. To do this, the data was divided into eighteen age groups: 0-5, 6-10, 11-15, 16-20, 21-25, 26-30, 31-35, 36-40, 41-45, 46-50, 51-55, 56-60, 61-65, 66-70, 71-75, 76-80, 81-85, ≥86 year.

First, it was tested whether the data set contains the same age effects as found in earlier conducted studies. This was done by dividing the data into two groups at the age thresholds of 50, 55, 60, 65, 70, 75 and 80 years old, and by dividing into four predefined life stages as <18, 18-39, 40-64 and ≥65. The analyses consisted of several analyses of variance (ANOVA) and *χ*^2^ tests. After showing that the age effects indeed exist when patients are divided as elderly and non-elderly and between life stages, the age groups were introduced to explore in detail the threshold point. To find out whether the numerical variables differ for neighboring age groups, multivariate analysis of variance (MANOVA) was used. This is a statistical procedure for comparing multivariate population means of several groups. It uses the variance-covariance between variables in testing the statistical significance of the mean differences [24]. Note that, since the analysis is not based on prediction models, ROC curves could not be used to select an optimal threshold. Before the analysis, it was made sure that the assumptions of independence of observations, random sampling, multivariate normality and homogeneity of variance and covariance are met.

## Results

### Characteristics of study subjects

**Fig 1:**
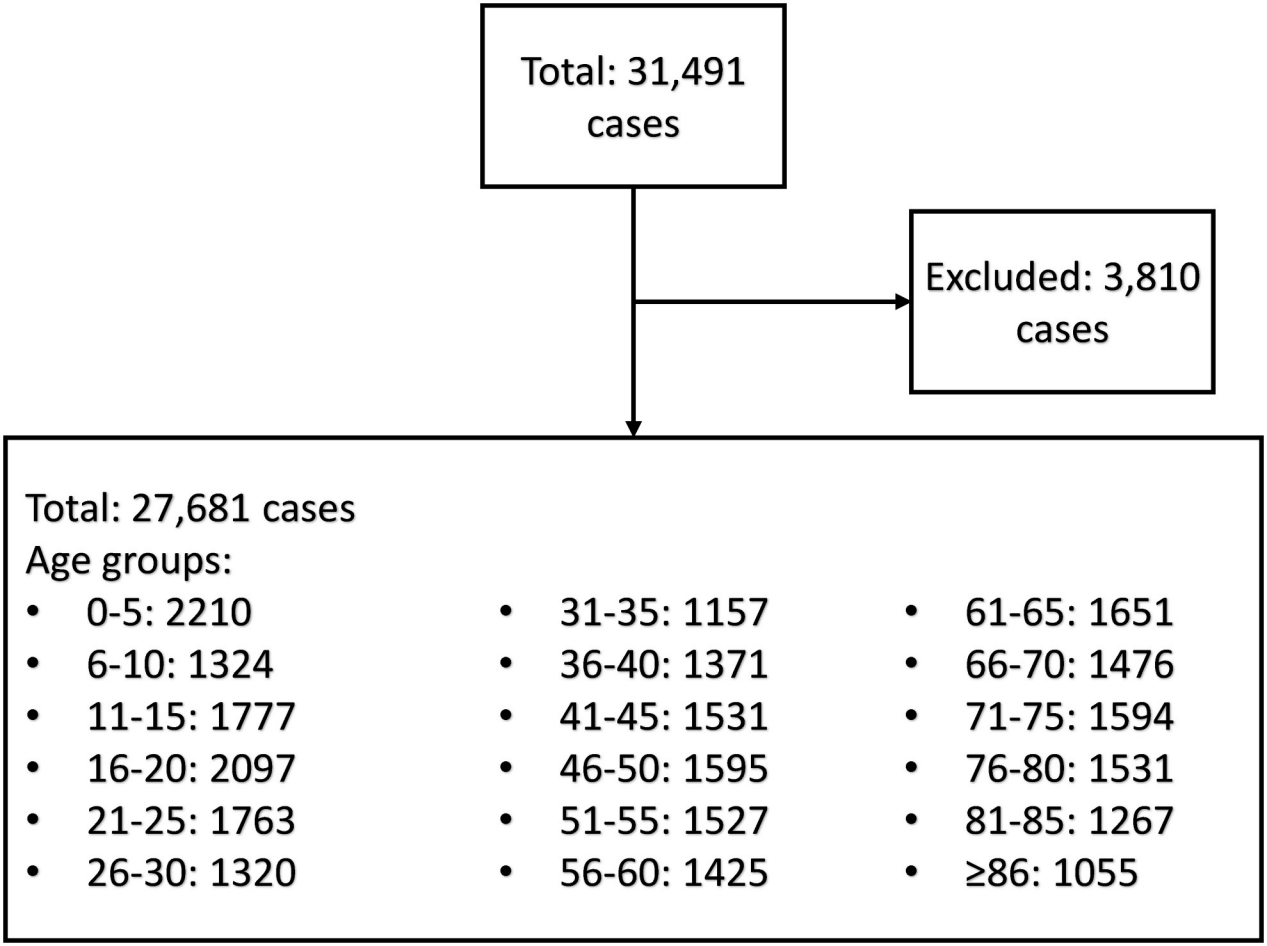
Distribution of the data set over the age groups. Excluded data include patients with a length of stay (LOS) of 0 seconds and patients coming in with bite or needle stick injuries.

In total, 31,491 ED visits were registered. After exclusion of needle and bite incidents and of patients directly transferred to other departments, a total of 27,681 ED visits were left for analysis. In this data set, 53.3% patients were male and 46.7% were female. Fig 1 depicts the distribution of the data set over the eighteen age groups.

The results of the missing value analysis indicate that missing values do not occur completely at random (MCAR). Yet due to the small proportion of missing values in each variable (less than 10%) [25] and the large size of the sample, we followed list-wise deletion method, as it is preferred over imputing missing values since imputation may bias significance and size of the effects [24].

### Main results

First, it is tested whether the effects found in previous literature also hold in our data set. This is done by splitting the data several times in two groups (at the age of 50, 55, 60, 65, 70, 75 and 80 years old). In Table 1 the comparison is shown between age group younger than 65 years and 65 years or older, an often used age threshold. All the characteristics differ significantly between these two groups (p<0.05). Similar results are found for all other age thresholds dividing the patients into two groups.

**Table 1:** Comparison of <65 and ≥65 years patients. P-values are obtained by performing *χ*^2^ tests for the categorical variables (triage, type of referral and destination) and independent t-tests for the other variables.

	Patients aged <65 years (N = 20758)	Patients aged ≥65 years (N = 6923)	P-value
Mean age in years (SD)	31.4 (19.678)	77.4 (7.334)	
Waiting time (SD)	13.7 minutes (15.6)	8.8 minutes (13.7)	0.00
Treatment time (SD)	80.3 minutes (59.5)	126.3 minutes (67.0)	0.00
Length of stay (SD)	99.5 minutes (57.1)	139.0 minutes (65.8)	0.00
Number of specialties (%)*			
1	19635 (94.6%)	6171 (89.1%)	0.00
>1	1095 (5.4%)	752 (10.9%)	
Number of procedures (SD)	0.77 (0.89)	1.34 (1.12)	0.00
Number of tests	0.97 (1.18)	2.04 (1.64)	0.00
Triage N (%)**			
Red	55 (0.3%)	68 (1.0%)	0.00
Orange	1677 (8.2%)	940 (13.8%)	
Yellow	5650 (27.8%)	3070 (45.1%)	
Green	12 968 (63.7%)	2724 (40.0%)	
% Ambulance arrival	7.59%	21.64%	0.00
% Medication	33.7%	42.7%	0.00
% Medical (vs. trauma)* N (%)	5816 (28.1%)	3649 (52.7%)	0.00
Type of referral N (%)			
GP	4953 (23.9%)	3672 (53.0%)	0.00
Ambulance	12 677 (61.1%)	2235 (32.3%)	
Medical Specialist	169 (0.8%)	73 (1.1%)	
Self-referral	2114 (10.2%)	649 (9.4%)	
Other	844 (4.1%)	294 (4.2%)	
Destination N (%)			
Home	6812 (32.8%)	885 (12.8%)	0.00
Home + follow-up	9725 (46.8%)	2029 (29.3%)	
Admission Acute	2714 (13.1%)	3212 (46.4%)	
Admission Unit	1452 (7.0%)	766 (11.1%)	
Admission ward	8 (0.0%)	20 (0.3%)	
Mortuary	41 (0.2%)	6 (0.1%)	
Left without being seen			

* Division of specialties:
Medical specialties: internal medicine, pulmonology, cardiology, neurology, psychiatry, rheumatology, gastroenterology and paediatrics. Trauma Specialties: general surgery, urology, plastic surgery, orthopaedics, gynaecology, ENT, ophthalmology, dermatology, stomatology

** Triage level: Red = most urgent, Green = least urgent

For the comparison with threshold 65 years older patients have shorter waiting times than the younger (means of ±9 minutes versus ±14 minutes), which might be dependent on their higher urgent triage levels. Once entering the treatment room, older patients tend to have longer treatment time than younger patients (means of ±126 minutes vs. ±80 minutes). The overall length of stay longer than that of younger patients (means of ±2 hours and 19 minutes vs. ±1 hour and 40 minutes). The number of specialties involved with the elderly patient’s treatment is higher (means of 1.11 specialties vs. 1.05 specialties), as well as the number of tests ordered (means of 2.04 tests vs. 0.97 tests) and the number of medical procedures performed (means of 1.34 vs. 0.77). Older patients visit different specialists on the ED than younger: their main specialty involved is more often (means of 52.7% vs. 33.6%) a medical than a surgical (trauma) specialty. Patients above the age of 65 are more likely to get medication administered during their stay on the ED (means of 42.3% vs. 33.6%) and have a higher chance of getting admitted to the Acute Admission Unit (AAU) whereas younger patients tend to go home with a follow-up appointment. With similar results are found for all other age thresholds, it can be concluded that the results of the analyses correspond with the results found in previous studies.

Table 2 shows the results for categorization according to life stages. Except for the waiting times of <18 and 18-39 groups, all variables differ significantly between each life stage similar to the comparison between elderly and non-elderly. The length of stay steadily increases with the higher life stages as a result of a longer treatment time for older patients although their waiting time decreases. The number of procedures, the number of tests applied and the number of specialties involved also shows a constant increase as the life stage increases which would also explain why the treatment time is higher.

**Table 2:** Comparison of the mean values of the variables for the different life stages (<18, 18-39, 40-64 and ≥65). Results are obtained by performing an ANOVA analysis, in which the mean values of each life stage are compared with the mean values of the other life stages.

	Life stage	Mean	Std. Deviation	N
Waiting time (in min)	<18	14.35*	15.79868	5596
	18-39	14.68*	15.81107	5921
	40-64	12.63	15.31159	7004
	≥ 65	8.84	13.76636	6734
	**Total**	**12.48**	**15.32607**	**25255**
LOS (in min)	<18	86.58	47.8467	5596
	18-39	90.5	56.30775	5921
	40-64	109.68	62.20314	7004
	≥ 65	137.69	63.92483	6734
	**Total**	**107.53**	**61.82174**	**25255**
Number of specialties	<18	1.04	0.197	5596
	18-39	1.05	0.223	5921
	40-64	1.07	0.25	7004
	≥ 65	1.11	0.31	6734
	**Total**	**1.07**	**0.253**	**25255**
Number of procedures	<18	0.61	0.781	5596
	18-39	0.76	0.84	5921
	40-64	0.96	0.978	7004
	≥ 65	1.37	1.121	6734
	**Total**	**0.94**	**0.991**	**25255**
Number of tests	<18	0.61	0.762	5596
	18-39	0.9	1.059	5921
	40-64	1.31	1.392	7004
	≥ 65	2.08	1.64	6734
	**Total**	**1.26**	**1.396**	**25255**
Treatment time (in min)	<18	69.13	48.613	5596
	18-39	72.6	56.114	5921
	40-64	93.42	62.733	7004
	≥ 65	125.48	65.144	6734
	**Total**	**91.7**	**63.151**	**25255**

* When comparing the means of each life stage with each other, the ANOVA analysis shows that the means of the waiting time for the age groups ‘<18 years’ and ‘18-39 years’ do not differ significantly. All other means are significantly different (P<0.05).

**Fig 2:**
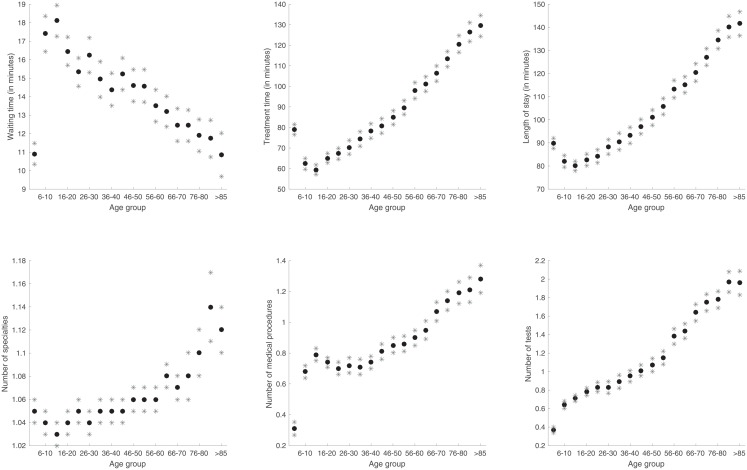
Graphical representations of the means of the variables length of stay, waiting time, treatment time, number of medical procedures performed, number of specialists involved in treatment and number of test performed for each age group. The asterisks indicate the boundaries of the 95% confidence intervals.

After establishing that the effects as reported in earlier studies indeed also exist in the data set of this study, we analyzed the data in more detail in order to seek a more precise threshold. The resulting graphs of the MANOVA applied to eighteen age groups for numerical variables are shown in Fig 2.

**Fig 3:**
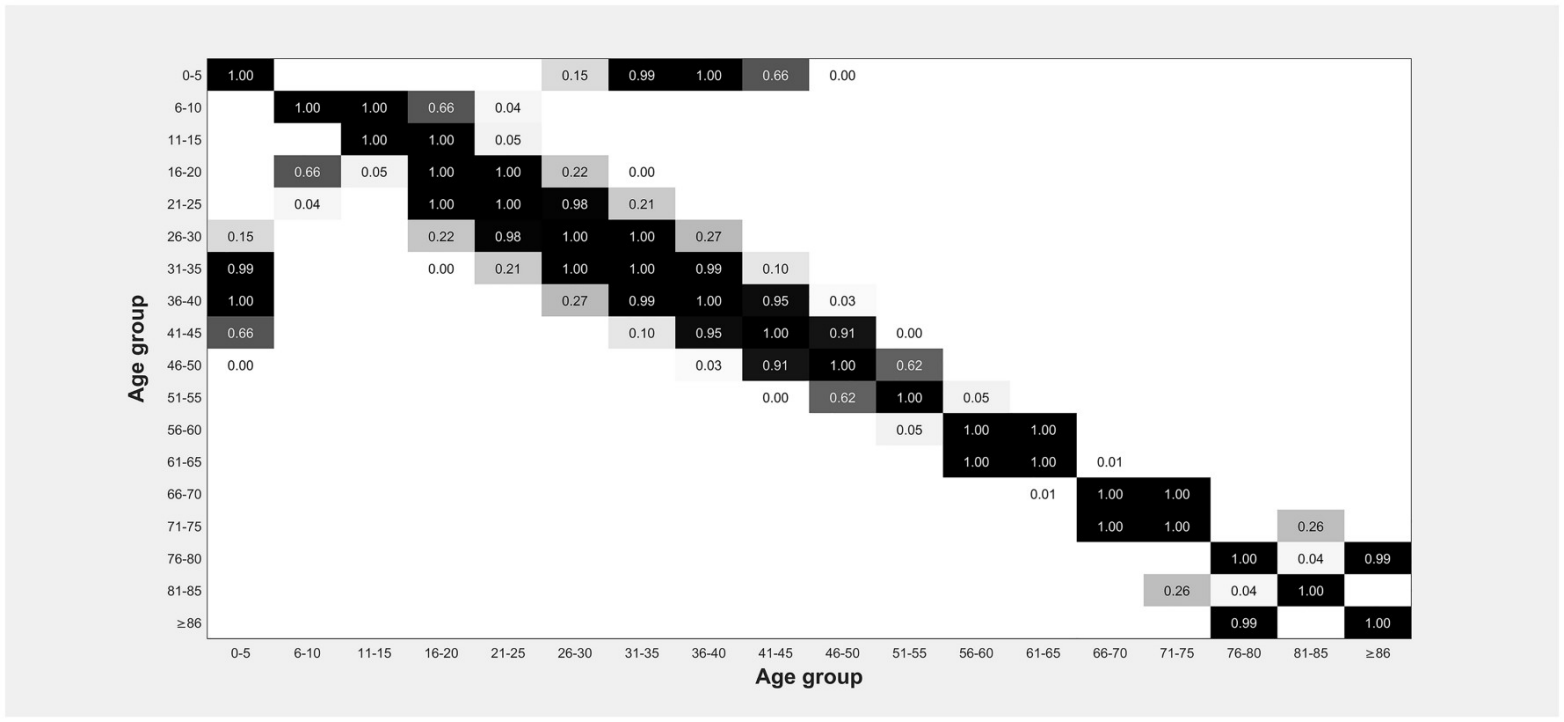
P-value matrix for the variable ‘treatment time’. Black cells indicate non-significant differences between age groups and significant difference with p = 0.000 are left out. The matrix shows that there are virtually no differences between the consecutive age groups for the considered variables.

The results of this analysis indicate that there are virtually no differences between the consecutive age groups for the considered variables. There were significant differences between a few consecutive groups, which shows that it is possible to have groups of patients who are close in age but still differ from each other. Yet this does not indicate a clear threshold for grouping the complete patient set since moving the threshold down or up also gives similar results. Combining p-value matrices (such as the one showed in Fig 3) reveals no clear pattern.

The patients are divided into groups of trauma or medical visits as well as triage categories and similar results are found for each of these patient groups. Therefore, we can conclude that these aspects do not cause the results we find in this study. When enlarging the age range of the age groups, we find that the patient characteristics of the age groups only start to differ significantly when they span at least ten years.

### Limitations

The results of the missing value analysis indicate that list-wise deletion method may have introduced some bias to the analysis since data is not missing completely at random. Yet there are controversial studies arguing that it should still be preferred over imputation, primarily for large data sets with relatively small missing ratios such as the current data set [24]. Also, the results are coherent with the findings of previous studies on elderly research [3, 5–11].

The study is based on data from one emergency room which may raise concerns in generalizing the results. It is also likely that the population visiting the Dutch emergency department differs from populations in other countries due to differences in the insurance policy and health care systems. However, the outcomes of this study are in line with those of the prior studies conducted in different countries [3, 5–11]. Therefore, it is plausible that these drawbacks do not notably affect the results.

## Discussion

This study examines the differences in patients according to age and aims to find whether a clear age threshold between young and old patients in the emergency department exists.

Almost none of the consecutive age group differences are significant in groupings of small-sized windows (e.g. 5 years) and almost all of them are significant when the size of the windows is increased. Therefore, although age has an effect on the variables measured on the ED, assigning a threshold that will divide the patients into two distinctive groups is not possible based on age factor only. We could not find support that the often-used age boundary of 65 years is better than any other, for example 60 or 70 years. The age threshold of 65 years might have been chosen in the context of socio-economic conventions. This study does not question the validity of the 65-threshold for other medical applications, but it shows that it is not observed within an ED treatment setting.

Confirming the results of prior studies, we find that the length of stay increases with age [2–4, 8, 11, 14]. Fayyaz and colleagues [5] also mention an increased use of ED resources for the elderly. We found this effect too when testing for the length of the treatment time, the number of tests, the number of medical treatment actions performed on patients, the medication use on the ED for patients and, to a lesser extent, the number of doctors involved in treating a patient. Baum and colleagues [6] found the same result as we did regarding the number of tests ordered: more tests were ordered for older patients than for the younger [2]. Several other researchers mention a higher hospital admission rate for elderly [2, 3, 7, 9, 13, 15, 16]. We find this higher rate in our results as well. When testing for the way the different patient groups enter the ED, we find that the elderly arrive at the ED more often by ambulance, like also reported by Baum, Strange, and Wofford and colleagues [2, 4, 6].

We also found that elderly patients are more likely to be referred to the ED, for example by a general practitioner, outpatient clinic or nursing ward. These results are not mentioned in other studies. Our results also show that the condition of elderly entering the ED is often more severe than that of younger patients as indicated by higher urgent triage categories. This result corresponds with the results of Aminzadeh and colleagues and Fayyaz and colleagues [3, 5]. This fact could be related to the shorter time elderly have to wait before being treated, which is found in this study but is not yet supported by other research.

For future research, we would suggest searching for other ways to divide the patient data into more natural groups instead of using chronological age only. This can be done by using a clustering method or by using models that take into account a fuzzy or gradual transition between the groups. Furthermore, replicating this study with data from different hospitals and more variables, such as the existence of co-morbidities, would improve the overall generalizability of the study. The results of this study suggest that a threshold based on chronological age on the ED might not be the most suitable choice and should be questioned. Although age has an effect on the emergency room treatment, the differentiation should not be done by a simple threshold, but by a more patient-centric approach that takes more variables into account.
